# New Grafted Copolymers Carrying Betaine Units Based on Gellan and N-Vinylimidazole as Precursors for Design of Drug Delivery Systems

**DOI:** 10.3390/molecules25225451

**Published:** 2020-11-20

**Authors:** Stefania Racovita, Nicolae Baranov, Ana Maria Macsim, Catalina Lionte, Corina Cheptea, Valeriu Sunel, Marcel Popa, Silvia Vasiliu, Jacques Desbrieres

**Affiliations:** 1 “Petru Poni” Institute of Macromolecular Chemistry, Grigore Ghica Voda Alley, No. 41A, 700487 Iasi, Romania; stefania.racovita@icmpp.ro (S.R.); macsim.ana@icmpp.ro (A.M.M.); silvia.vasiliu@icmpp.ro (S.V.); 2 Department of Natural and Synthetic Polymers, Faculty of Chemical Engienering and Environmental Protection, “Gheorghe Asachi” Technical University of Iasi, Prof. Dr. Docent Dimitrie Mangeron Street, No. 73, 700050 Iasi, Romania; Baranov_nicolae@yahoo.com (N.B.); marpopa2001@yahoo.fr (M.P.); 3 Faculty of Chemistry, “Al. I. Cuza” University, Carol 1 Bvd., No. 11, 700506 Iasi, Romania; vsunel@uaic.ro; 4 Faculty of Medicine, “Gr. T. Popa” University of Medicine and Pharmacy, Universitatii Street, No.16, 700115 Iasi, Romania; clionte@yahoo.com; 5 Department of Biomedical Sciences, Faculty of Biomedical Bioengineering, “Gr. T. Popa” University of Medicine and Pharmacy, Kogalniceanu Street No. 9-13, 700454 Iasi, Romania; coricheptea@yahoo.com; 6 Academy of Romanian Scientists, Splaiul Independentei Street No. 54, 050085 Bucuresti, Romania; 7 Institut des Sciences Analytiques et de Physico-Chimie Pour l’Environnement et les Materiaux (IPREM), Pau and Pays de l’Adour University (UPPA), UMR CNRS 5254, Helioparc Pau Pyrenees, 2, av. President Angot, 64053 Pau CEDEX 09, France

**Keywords:** graft polymerization, *N*-vinylimidazole, gellan gum, betaine structure

## Abstract

New grafted copolymers possessing structural units of 1-vinyl-3-(1-carboxymethyl) imidazolium betaine were obtained by graft copolymerization of *N*-vinylimidazole onto gellan gum followed by the polymer-analogous reactions on grafted polymer with the highest grafting percentage using sodium chloroacetate as the betainization agent. The grafted copolymers were prepared using ammonium persulfate/*N*,*N*,*N*′,*N*′ tetramethylethylenediamine in a nitrogen atmosphere. The grafting reaction conditions were optimized by changing one of the following reaction parameters: initiator concentration, monomer concentration, polymer concentration, reaction time or temperature, while the other parameters remained constant. The highest grafting yield was obtained under the following reaction conditions: c_i_ = 0.08 mol/L, c_m_ = 0.8 mol/L, c_p_ = 8 g/L, t_r_ = 4 h and T = 50 °C. The kinetics of the graft copolymerization of *N*-vinylimidazole onto gellan was discussed and a suitable reaction mechanism was proposed. The evidence of the grafting reaction was confirmed through FTIR spectroscopy, X-ray diffraction, ^1^H-NMR spectroscopy and scanning electron microscopy. The grafted copolymer with betaine structure was obtained by a nucleophilic substitution reaction where the betainization agent was sodium chloroacetate. Preliminary results prove the ability of the grafted copolymers to bind amphoteric drugs (cefotaxime) and, therefore, the possibility of developing the new sustained drug release systems.

## 1. Introduction

In recent years, the chemical modification of natural polymers, polysaccharides in particular, by graft polymerization or by introduction of some functional groups, represents one of the most accesible and attractive method to obtain the polymeric materials with desired properties [1].

Since 1978, when gellan gum (GLL) was isolated for the first time from *Pseudomonas elodea*, this microbial polysaccharide has been used in various applications, such as in food industry as a thickening agent [2] or in biomedical and pharmaceutical fields [3,4].

This polysaccharide can be chemically modified due to the presence of many hydroxyl groups that can act as possible sites for grafting reaction. In the literature, some studies were found regarding the grafting of different vinyl monomers onto gellan gum chains using various sources of initiator radicals such as persulfate, redox systems or microwave irradiations [5,6]. Acrylic and methacrylic monomers such as, acrylamide [7], methacrylamide [8], *N,N*-dimethylacrylamide [5] or 2-(dimethylamino)ethyl methacrylate [9] were grafted onto gellan backbone in order to obtain polymeric materials with applications in industry as adsorbents/flocculants or in pharmaceutical field as sustained/controlled drug delivery systems. Verma et al. [10] combined the properties of *N*-vinyl-2-pyrrolidone and gellan gum by a grafting reaction, obtaining a new product that can be used as a coating material, superadsorbent or flocculating agent in the mining industry.

Among the vinyl monomers, *N*-vinylimidazole (NVI) has gained much attention in past years because the grafted polymers containing the imidazole ring are biocompatible, biodegradable and show antibacterial activity [11,12,13,14,15]. They were successfully used in medical and pharmaceutical fields. Moreover, chemical modification of imidazole-based polymers opens a huge opportunity for the development of functional polymers with complex structures [16].

The term polymers carrying betaine units (PB), or polyzwitterions, refers to polymers containing both negative (carboxylate group, sulfonate group or phosphonate/phosphate/phosphinate group) and positive (*onium* group without hydrogen atoms) charges located in the same repeat unit and separated by an alkylene group [17,18,19,20].

The grafted polymer carrying betaine units (PGB) can be obtained by one step and multistep processes [21,22].

One step process can be achieved by grafting monomers with a betaine structure onto the polymer backbone, while the multistep process consists of grafting monomers containing both nitrogen and vinyl group followed by polymer-analogous reactions onto grafted polymers in the presence of betainization agents.

The commercial interest for these polymers is determined by their possibility to be used as polymeric sorbents, oil recovery agents, fungicides, flame retardant polymers, emulsifying agents, wetting agents, cleaning agents and cryoprotectants [23,24,25]. Polymers having various betaine structure were synthesized in order to obtain materials sensitive to different stimuli (pH, temperature, etc.) [26].

The most interesting properties of PB are bio and hemocompatibility. Various studies have shown that polymer coatings based on poly(phosphobetaine) can improve the biocompatibility of some ocular devices by decreasing the adhesion of some microorganisms and eukaryotic cells [27,28]. Poly (methyl methacrylate) discs coated with a layer of sulfobetainic copolymer were exposed to *Staphylococcus epidermidis, Staphylococcus aureus* and *Pseudomonas aeruginosa* and it was found that the number of bacteria adhering to the surface of the discs coated with a layer of polybetaine decreased compared to the uncovered discs [29].

Due to the presence of vinyl groups capable of grafting polymerization and tertiary nitrogen suitable for the polymer-analogous reactions, the *N*-vinylimidazole is a very good candidate to obtain polymers carrying betaine units [30,31].

In this context, the work described in this paper follows several aspects: (1) synthesis of new grafted copolymers starting from gellan gum and *N*-vinylimidazole (PG); (2) finding the optimal conditions of the grafting reaction; (3) evidence of the grafting reaction by different methods; (4) chemical modification of new grafted copolymers containing an imidazole ring with the highest grafting yield by polymer analogous reactions in order to obtain the grafted copolymer with a betaine structure (PGB1); (5) immobilization and drug release studies of an antibiotic drug.

## 2. Results and Discussion

The preparation of grafted copolymers with betaine structure took place in two steps.

**Figure 1:**
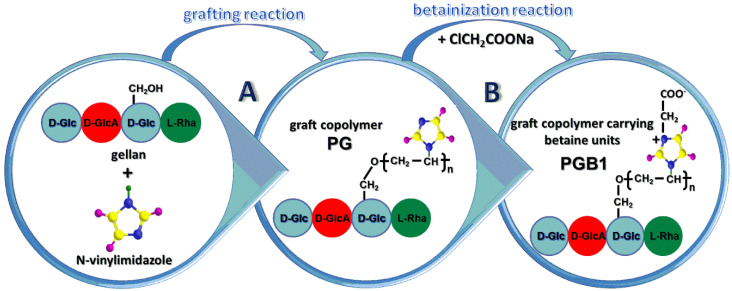
Graphical representation of the synthesis pathway of PG and grafted polymer carrying betaine units (PGB1) copolymers.

1. Preparation of PG and finding the optimal conditions for obtaining the grafted copolymer with maximum grafting yield.
2. Betainization reaction of PG with the highest grafting yield in the presence of sodium chloroacetate (Figure 1).

### 2.1. Optimal Conditions for the Preparation of PG

Various parameters such as monomer, initiator and polymer concentration, temperature and reaction time were investigated in order to optimize the reaction conditions in graft copolymerization of NVI onto gellan gum, as well as to improve the copolymer yield. Optimal reaction conditions were found by modifying one of the above-mentioned reaction parameters keeping the others constant, the reaction taking place under vigorous stirring and under a N_2_ atmosphere.

#### 2.1.1. Influence of Initiator Concentration

**Figure 2:**
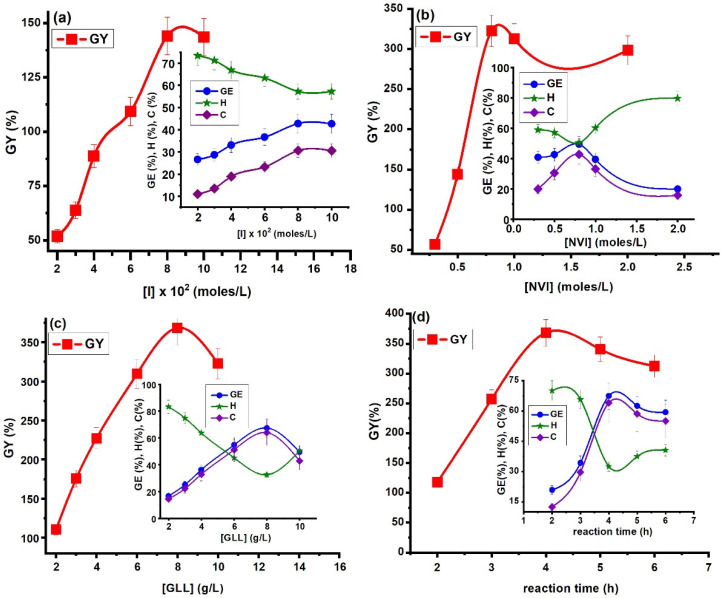
Influence of various factors on grafting parameters: (**a**) initiator concentration (c_m_ = 0.5 mol/L; c_p_ = 10 g/L, T = 60 °C and t = 4 h); (**b**) monomer concentration (c_i_ = 0.08 mol/L, c_p_ = 10 g/L, T = 60 °C and t_r_ = 4 h); (**c**) gellan concentration (c_i_ = 0.08 mol/L; c_m_ = 0.8 mol/L; T = 60 °C and t_r_ = 4 h); (**d**) reaction time (c_i_ = 0.08 mol/L; c_m_ = 0.8 mol/L; c_p_ = 8 g/L; T = 60 °C).

In the initiation stage, the rate of grafting can be influenced by a number of factors such as the nature and the concentration of the initiator, as well as the solubility of the initiator in the reaction medium. Among the thermal initiators, ammonium persulfate (APS) is preferred due to its high solubilization in water. In this study a redox initiator APS and *N*,*N*,*N*′,*N*′ tetramethylethylenediamine (TEMED) (molar ratio 1:1) was used, and the influence of the initiator concentration on the grafting parameters was performed by modifying the APS/TEMED concentration in the range of 0.02–0.1 mol/L, keeping the monomer and gellan concentrations (c_m_ = 0.5 mol/L; c_p_ = 10 g/L), temperature (T = 60 °C) and reaction time (t = 4 h) constant. The graphical representation of grafting parameters versus initiator concentration is presented in Figure 2a.

As can be seen from Figure 2a grafting parameters like grafting yield (GY, %), grafting efficiency (GE, %) and conversion (C, %) increase with initiator concentration, reaching the maximal value at 0.08 mol/L APS/TEMED. After that, a slight decrease of grafting parameters is observed. This behavior could be explained in the following manner: at the beginning of the initiation reaction, the number of active sites on the gellan chain increases with the increase of initiator concentration, leading both to the formation of gellan macroradicals as well as the initiation of the grafting reaction. Then, at an initiator concentration greater than 0.08 mol/L, a decrease of grafting parameter values is observed probably because of an increase in the rate of homopolymerization compared to the grafting rate, this observation being proven by the increase in the value of homopolymer yield (H%). Another explanation of the decrease of grafting parameters can be attributed to a competition between initiation and termination reactions, the latter being achieved through chain-transfer to initiator or by a coupling reaction between initiator radicals [32]. The same behaviors were observed in the case of graft copolymerization of *N*-vinylimidazole onto various polysaccharides: xanthan [14], hyaluronic acid [11] and carboxymethyl starch [32].

#### 2.1.2. Influence of Monomer Concentration

Regarding the influence of the monomer concentration on the grafting yield, there are several factors that must be taken into account: monomer reactivity, polarity, steric hindrance, stability of the monomer radicals and concentration of monomer. In this study, the monomer concentration was varied within the range 0.3–2 mol/L, keeping the other parameters constant: c_i_ = 0.08 mol/L, c_p_ = 10 g/L, T = 60 °C and t_r_ = 4 h. The influence of NVI concentration on the grafting reaction is illustrated in Figure 2b. The results revealed an increase of GY%, GE% and C% with increasing NVI concentration up to 0.8 mol/L due to the greater availability of NVI molecules in the immediate vicinity of active sites located on the gellan chain, leading both to chain initiation and the formation of free radical donor that participate in the propagation reaction.

Thereafter, an increase in the monomer concentration above 0.8 mol/L caused a decrease of grafting parameter values, an exception being observed for the H% values that were found to increase. This behavior can be explained on the one hand by degradative chain-transfer to NVI as proposed in literature by Bamford and Schofield [33], and on the other hand by the poor diffusion of the monomer to the active sites situated on the polysaccharide chains leading to the homopolymer formation. A similar trend was reported in the literature for grafting of various vinyl monomers onto polysaccharides [11,32].

#### 2.1.3. Influence of Gellan Concentration

The influence of polysaccharide concentration on grafting parameters is shown in Figure 2c. The gellan concentration was modified in the range 2–10 g/L, keeping constant the other parameters: c_i_ = 0.08 mol/L; c_m_ = 0.8 mol/L; T = 60 °C and t_r_ = 4 h. As can be seen from Figure 2c the grafting parameters (GY%, GE% and C%) increased with increasing of gellan concentration until 8 g/L and then a decrease of these parameters was observed. The increase of the grafting parameters until c_p_ = 8 g/L could be attributed to the increased number of grafting active centers situated on the polysaccharide chains leading to the formation of graft copolymers. An increase of polymer concentration above 8 g/L determines the increase of the viscosity of the reaction medium which hinders the possibility of e NVI movement toward the active sites situated on the gellan chains. The same behavior was observed in case of grafting on *N*,*N*-dimethylacrylamide onto gellan gum [5].

#### 2.1.4. Influence of Reaction Time

The influence of reaction time on the grafting parameters is presented in Figure 2d and was determined by changing the time period of grafting reaction from 2 h to 6 h, keeping all the other reaction parameters constant (c_i_ = 0.08 mol/L; c_m_ = 0.8 mol/L; c_p_ = 8 g/L; T = 60 °C).

The obtained results revealed that the values of grafting parameters (GY%, GE% and C%) increased with increasing reaction time up to 4 h, due to the increase of active sites number situated on the gellan chains. After that, an increase in reaction time led to a slow decrease of the values of the grafting parameters. However, there was a slight increase in the homopolymer yield, suggesting the preference of the monomer to its own macroradical than for the one on the grafted chain, probably due to some steric hindrances. The same behavior was observed by other investigators whose results have been published in the literature [34,35].

#### 2.1.5. Influence of Temperature

**Figure 3:**
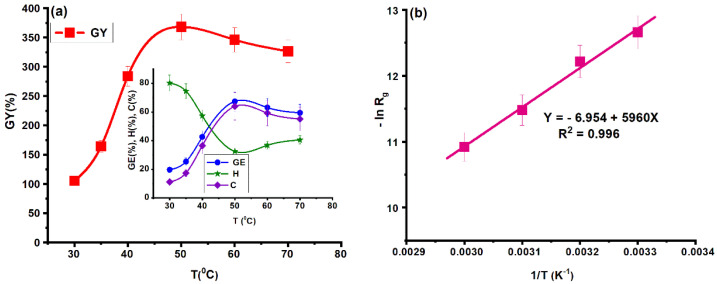
Influence of temperature on grafting of *N*-vinylimidazole (NVI) onto gellan gum (**a**) (c_i_ = 0.08 mol/L; c_m_ = 0.8 mol/L; c_p_ = 8 g/L and t_r_ = 4 h) and (**b**) the activation energy of the grafting reaction.

Temperature is one of the most important factors that influence the kinetics of graft copolymerization. The effect of temperature on the grafting parameters is shown in Figure 3a.

Temperature was varied from 30 to 80 °C, keeping constant c_i_ = 0.08 mol/L; c_m_ = 0.8 mol/L; c_p_ = 8 g/L and t_r_ = 4 h. As can be observed from Figure 3a the optimal temperature for maximum grafting yield was 50 °C and increasing temperature led to enhancement of the grafting parameters. The increase of grafting parameters until 50 °C can be attributed to:

(1) an increase in the production of free radicals resulting from thermal decomposition of initiator leading to increase in the number of the active sites on the polysaccharide chains;
(2) a decrease of reaction medium viscosity;
(3) fast diffusion of the monomer toward the active sites from the polysaccharide backbone;
(4) increase in rate of initiation and propagation steps [13,36].

Above 50 °C, the values of the grafting parameters decreased, probably due to the increase of the rate of the termination step by a combination of the monomer radicals and formation of the homopolymer. The effect may also have been due to the slower diffusion of the monomer to growing grafts due to steric hindrances, and its preference for growing homopolymer macroradicals.

The activation energy is another important parameter that can give information about the grafting process and can be determined from Arrhenius equation, as follows:

(1) $$k=A\cdot e^{-E_{a}/RT}$$

where *k* is the rate constant, *A* is the pre-exponential factor, *R* is the gas constant (8.314 J·K^−1^·mol^−1^) and *T* is the absolute temperature (K).

The general rate equation of the grafting reaction can be written:

(2) $$R_{g}=A\cdot {\left[I\right]}^{a}\cdot {\left[M\right]}^{b}\cdot {\left[P\right]}^{c}\cdot e^{-E_{a}/RT}$$

(3) $$lnR_{g}=lnk^{\prime }-\frac{E_{a}}{RT}$$

(4) $$k^{\prime }=A\cdot {\left[I\right]}^{a}\cdot {\left[M\right]}^{b}\cdot {\left[P\right]}^{c}$$

where [*I*] = initiator concentration; [*M*] = monomer concentration; [*P*] = polymer concentration; *a*, *b*, and *c*—reaction order with respect to initiator, monomer and polymer.

The slope (−*E_a_*/*RT*) of the *lnR_g_* plots versus 1/*T* is used to find the *E_a_* value (Figure 3b). The activation energy for PG was 49.5 KJ/mol. Similar results were observed in the case of grafting of various vinyl monomers (acrylamide, acrylic acid and methyl methacrylate) onto starch [37].

From the above studies it can be said that the optimized reaction conditions for grafting reaction of *N*-vinylimidazole onto gellan gum are as follows: initiator concentration = 0.08 mol/L; monomer concentration = 0.8 mol/L; polymer concentration = 8 g/L, reaction temperature = 50 °C and reaction time = 4 h.

### 2.2. Kinetics and Mechanism of graft Copolymerization

Generally, the rate equation of radical polymerization reaction is:

(5) $$R_{g}=k\cdot {\left[I\right]}^{1/2}\cdot \left[M\right]\cdot {\left[P\right]}^{1/2}$$

The rate of graft copolymerization depends on several parameters such as initiator, monomer and polymer concentrations. In this study the graft copolymerization of *NVI* onto gellan was studied by modifying one of the parameters while the other parameters remained constant. In this context, the rate equations are:

(6) $$lnR_{g}=lnk_{1}+a\cdot ln\left[I\right],\;k_{1}=K\cdot {\left[M\right]}^{b}\cdot {\left[P\right]}^{c}$$

(7) $$lnR_{g}=lnk_{2}+b\cdot ln\left[M\right],\;k_{2}=K\cdot {\left[I\right]}^{a}\cdot {\left[P\right]}^{c}$$

(8) $$lnR_{g}=lnk_{3}+c\cdot ln\left[P\right],\;k_{3}=K\cdot {\left[I\right]}^{a}\cdot {\left[M\right]}^{b}$$

**Figure 4:**
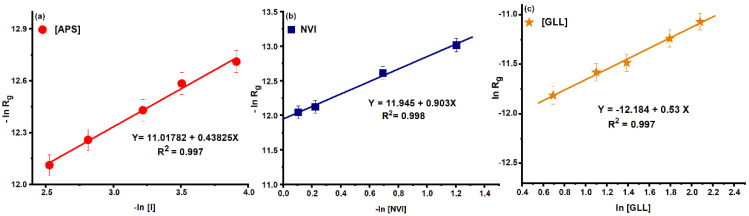
Plotting *lnR_g_* versus (**a**) *ln* [*I*]; (**b**) *ln* [*NVI*]; and (**c**) *ln* [*GLL*].

The graphical representations of *lnR_g_* versus *ln*[*I*], *ln*[*M*] and *ln*[*P*], respectively represent a straight line and are shown in Figure 4.

From Figure 4 the values of the slopes of the plots suggest that the reaction order with respect to initiator, monomer and polymer are 0.44, 0.90 and 0.53, respectively. Thus, the rate equation of grafting reaction of *N*-vinylimidazole onto gellan gum is:

(9) $$R_{g}=k\cdot {\left[APS/TEMED\right]}^{0.44}\cdot {\left[NVI\right]}^{0.903}\cdot {\left[GLL\right]}^{0.53}$$

Equation (13) is very similar to Equation (9) and, for this reason, the mechanism of the grafting reaction of NVI onto gellan involves the same elementary reactions encountered in the free radical polymerization, namely, initiation, chain growth or chain propagation, chain transfer and termination of polymer chains [38].

**Figure 5:**
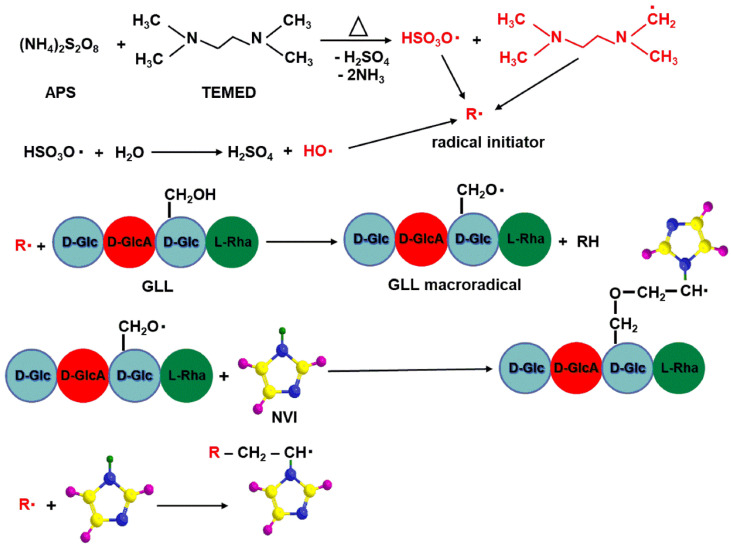
Initiation step of grafting copolymerization.

The first step of the radical polymerization reaction corresponds to the formation of active species (radicals) followed by their addition to the monomer molecule (Figure 5).

**Figure 6:**
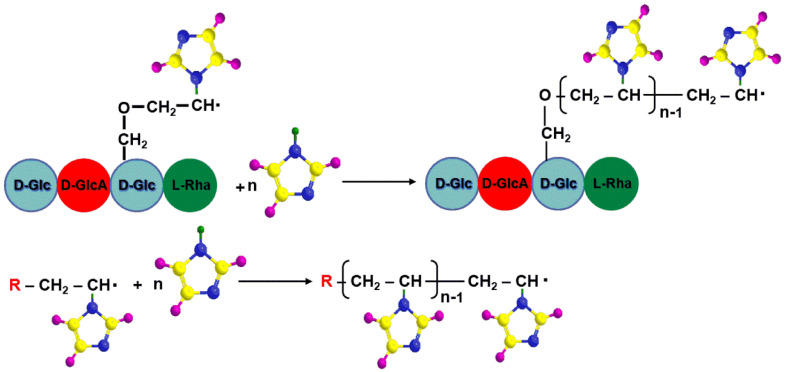
Propagation step of grafting copolymerization.

When the initiation system is heated, it decomposes in radical species that are able to initiate the polymerization reaction. In the case of the APS and TEMED system, two types of radicals (alkylaminoethyl radical derived from TEMED and sulfate radical from APS) [39] are formed and are able to initiate the graft copolymerization of *N*-vinylimidazole onto the gellan backbone. The propagation step consists of successive addition of a large number of monomer molecule to the promoter generated in the initial step (Figure 6).

**Figure 7:**
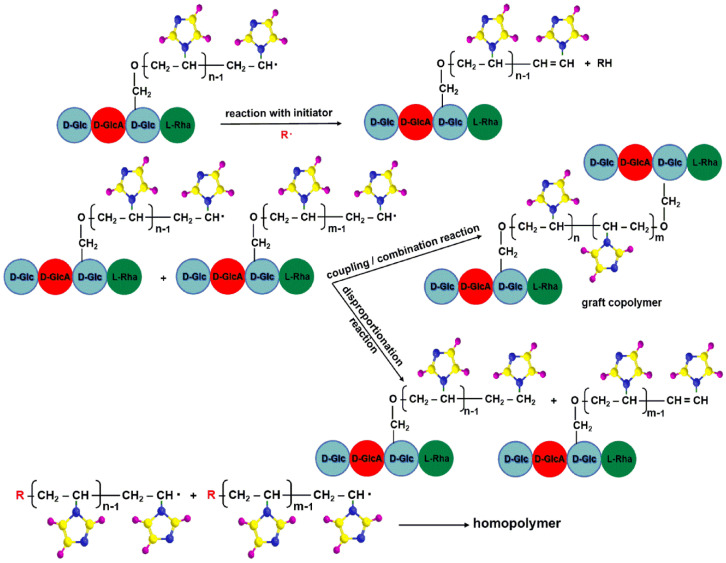
Termination steps of graft copolymerization.

The termination reactions depend on the size, activity and structure of the macroradical, the viscosity of the medium, the temperature and the composition of the reaction mixture (Figure 7). Depending on these factors, the mechanism of the termination reaction is different and occurs as follows: (a) reaction with the initiator; (b) coupling/combination and disproportionation reaction; (c) inactivation of the growth radicals by inhibitors.

### 2.3. Synthesis of Copolymer Carrying Betaine Structure

**Figure 8:**
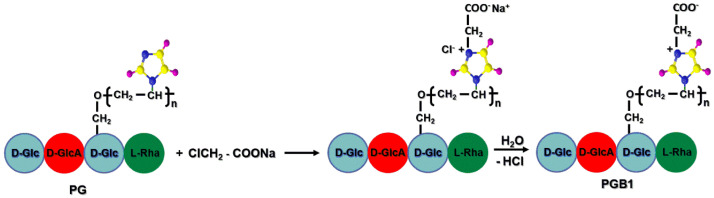
Betainization reaction of PG copolymer with sodium chloroacetate.

The second stage for preparation of grafted copolymer carrying structural units of 1-vinyl-3(1-carboxymethyl) imidazolium betaine consists in polymer-analogous reactions on grafted copolymer with the highest grafting yield in the presence of sodium chloroacetate as a betainization agent. From the reaction mechanism point of view, the grafted betaine copolymer with one methylene group between the opposite charges is achieved by the nucleophilic substitution reaction. The chemical structure of PGB1 is presented in Figure 8.

Different reaction parameters (temperature, reaction time, concentration of betainization agent) were modified in order to find the optimal value of betainization degree. It was found that the increase of all parameters mentioned above, up to a certain value, led to an increase of betainization degree. The optimal conditions for synthesis of graft copolymers carrying betaine structure was found to be: concentration of betainization degree = 20%, T = 60 °C and the reaction time = 72 h.

The degree of betainization was obtained by FTIR spectroscopy using the relative ratio between integrated area of betainized (1637 cm^−1^) and chemically unmodified imidazole ring (1500 cm^−1^). The curve fitting of the FTIR spectrum of PGB1 in the region 1700–1420 cm^−1^ was realized using OPUS Software on the basis of a linear regression Levenberg-Marquardt model, the method being similar to that mentioned in our previous paper [40]. In this case, the betainization degree was found to be 89.97%.

### 2.4. Characterization of PG and PGB1

#### 2.4.1. ^1^H-NMR Spectroscopy

**Figure 9:**
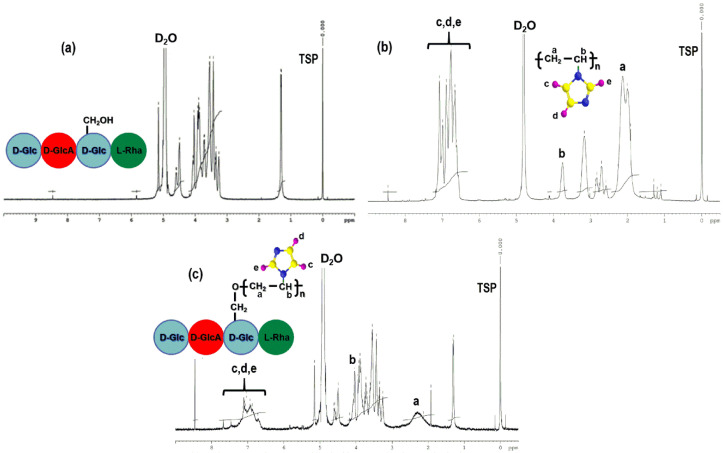
^1^H-NMR spectra of GLL (**a**); PNVI (**b**) and PG samples (**c**).

^1^H-NMR spectroscopy was used to elucidate only the structure of PG because the PGB1 is insoluble in the solvent used for PG, poly(*N*-vinylimidazole) (PNVI) and GLL samples. The structure of PG was elucidated by comparing the PNVI and GLL spectra with the spectrum of the grafting copolymer (Figure 9).

It is well known that for PNVI (Figure 9b) the following characteristic signals can be observed:

(1) multiplet signals at δ = 6.61–7.08 ppm assigned to the protons (H_2_, H_4_ and H_5_) belonging to the imidazole ring; (2) multiplet signals at δ = 3.7–3.9 ppm due to the methine protons; (3) doublet signal at δ = 2.07–2.13 ppm assigned to the backbone methylene protons; (4) triplet signal at δ = 2.58–2.87 ppm related to the splitting chain -CH group (isotactic, heterotactic and syndiotactic triads) [41].

The spectrum of GLL (Figure 9a) contains characteristic peaks of the tetrasaccharide repeating units as follows: -CH of glycosidic bonds in sugar at δ = 5.83 ppm; -CH of rhamnose at δ = 5.15 ppm; -CH of glucose and glucuronic acid at δ = 3.36–4.61 ppm; -CH_3_ of rhamnose at δ = 1.29–1.31 ppm [42,43].

The ^1^H-NMR spectrum presented in Figure 9c proved the synthesis of the PG copolymer because in this spectrum the characteristic signals of both PNVI (δ = 6.72–7.13 ppm—protons belonging to the imidazole ring; δ = 2.14–2.35 ppm—backbone methylene protons) and GLL (δ = 3.37–4.61 ppm—protons of the tetrasaccharide repeating units; δ = 1.29–1.31—CH_3_ of rhamnose) polymers are found.

#### 2.4.2. FTIR Spectroscopy

**Figure 10:**
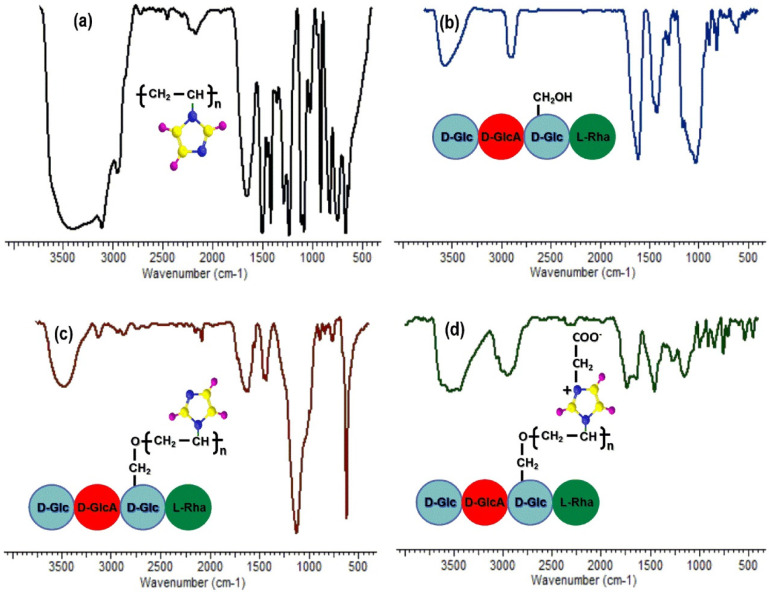
The infrared spectra of PNVI (**a**); GLL (**b**); PG (**c**) and PGB1 samples (**d**).

The infrared spectra of PNVI, GLL PG and PGB1 samples are presented in Figure 10.

The infrared spectrum analysis of gellan gum (Figure 10b) showed the following adsorption bands: 3566 cm^−1^ attributed to the O-H stretching of hydroxyl groups of glucopyranose ring; 2892 cm^−1^ characteristic of the aliphatic-CH; 1611 and 1420 cm^−1^ assigned to the asymmetric and symmetric vibrations of the carboxylate group and 1027 cm^−1^ attributed to the C-O-C bonds.

In the PNVI spectrum (Figure 10a) the following characteristic absorption bands are observed: 3100 cm^−1^ assigned to C-H stretching vibration; 2957 cm^−1^ attributed to the C-H and CH_2_ stretching vibrations of the backbone chain. A strong and broad band is observed at 1655 cm^−1^ that is characteristic of the C=C ring stretching vibrations. The bands observed at 1500, 1285, 1230, 1083 and 914 cm^−1^ are assigned to C-C, C=N ring stretching vibrations, C-H bending vibration, the ring C-H bending and C-C-C bending vibration of the backbone of the aliphatic chain. Two characteristic bands for the imidazole ring were observed at 1500 and 665 cm^−1^, the last being attributed to the puckering vibration of imidazole ring [41].

In the FT-IR spectrum of the PG copolymer (Figure 10c) a shifting of -OH stretching vibration from about 3400 cm^−1^ (3392 cm^−1^ for PNVI and 3415 cm^−1^ for GLL) to 3479 cm^−1^ is observed, as well as a decrease of intensity of the absorption bands corresponding to the -OH group vibration, indicating the participation of hydroxyl groups in the grafting reaction. The bands at 1630 cm^−1^ (>C=C< stretching vibrations in imidazole ring), 1545 cm^−1^ (>C=N^−^ stretching vibration) and 1124 cm^−1^ (in-plane bending vibration of the C-H bond inside imidazole ring) indicate the presence of PNVI in the structure of the grafted copolymer, and also the confirmation of grafting of the vinyl monomer onto the gellan backbone.

If the FT-IR spectra of PG and PGB1 copolymers are compared (Figure 10d) the appearance of new absorption bands specific to the betaine structure can be observed as follows: the band at 3439 cm^−1^ appears due to the >C=N^+^ group, and the shift of the adsorption band from 1630 cm^−1^ to 1637 cm^−1^ is due to the overlap the vibration band of >C=N^−^ group from PNVI with the absorption band corresponding to the asymmetric COO^−^ group. The absorption bands at 1340 and 1266 cm^−1^ may be attributed to the stretching vibration of carboxylate group.

#### 2.4.3. Surface Morphology Analysis

**Figure 11:**
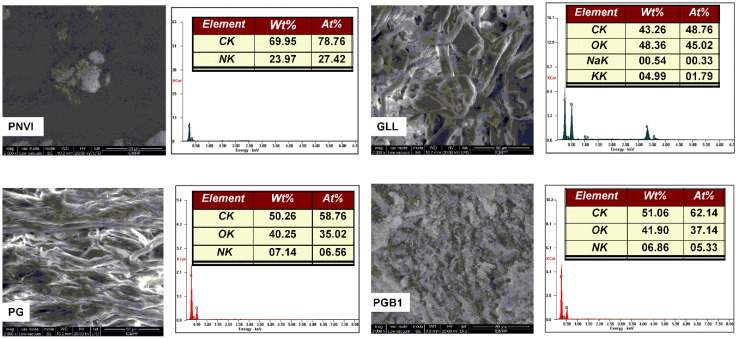
Scanning electron microscope (SEM) images and energy dispersive X-ray (EDAX) analysis of PNVI, GLL, PG and PGB1 samples.

Surface morphology images at high magnification (2000×) of PNVI, GLL, PG and PGB1 samples were analyzed using a scanning electron microscope coupled with an energy dispersive X-ray system and are presented in Figure 11.

The PNVI sample showed a compact porous structure while GLL and PG samples seemed to have a fibrous structure. When NVI was grafted onto the GLL backbone, microstructural changes were observed, the PG copolymer having a more ordered structure compared to that of the gellan. The reaction between PG copolymer and sodium chloroacetate led to the preparation of the grafting copolymer with betaine structure having a porous structure.

The EDAX analysis confirmed both grafting of *N*-vinylimidazole onto gellan gum and the formation of graft copolymer carrying betaine units as follows:

(1) the presence of nitrogen belonging only to NVI on the surface of PG and PGB1 copolymers;
(2) increase of C% value of the PG molecule compared to that from GLL indicating the presence of PNVI in the PG structure;
(3) increase of C% and O% values, as well as the decrease of N% values, of the PGB1 copolymer compared those from the PG copolymer due to the formation of betaine units.

#### 2.4.4. X-Ray Diffractions Analysis (XRD)

**Figure 12:**
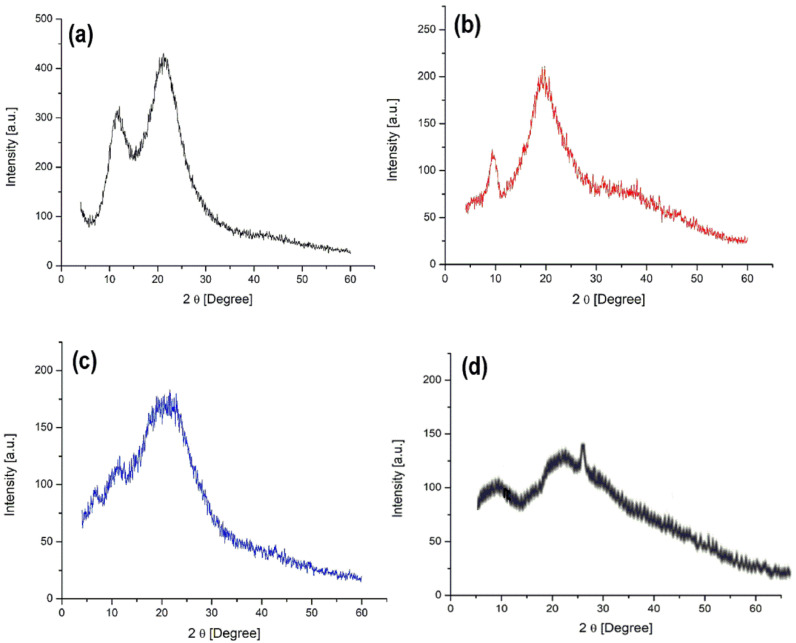
XRD patterns of PNVI (**a**); GLL (**b**); PG (**c**) and PGB1 samples (**d**).

XRD patterns of GLL, PNVI, PG and PGB1 samples are illustrated in Figure 12.

X-ray diffraction spectra of PNVI and GLL show two broad diffraction peaks at lower diffraction angle values (2θ = 20° and 10°) indicating the amorphous structure of both polymers. The XRD pattern of PG copolymer presents the combined signals of started polymers leading to the conclusion that the grafting of PNVI onto gellan gum was successfully completed. The PGB1 copolymers presented an amorphous structure.

#### 2.4.5. Thermogravimetric Studies

Thermogravimetric studies were realized to prove the preparation of both grafted copolymers and grafted copolymers carrying betaine units. The thermal stability of PNVI, GLL, PG and PGB1 samples was discussed according to the temperature at which intensive degradation took place and the results are presented in Table 1.

**Figure 13:**
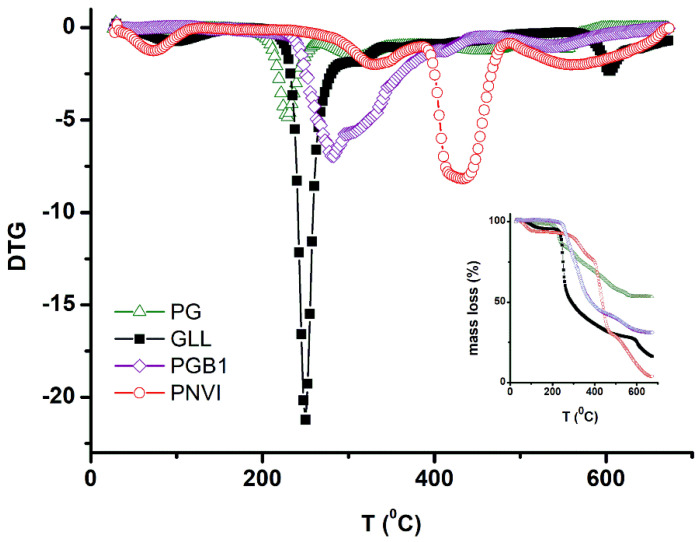
TG and DTG curves of PNVI, GLL, PG and PGB1 samples.

Thermogravimetric (TG) and derivative thermogravimetric (DTG) curves of PNVI, GLL, PG and PGB1 samples (Figure 13) showed that thermal degradation was characterized by four (PNVI), three (GLL), five (PG) and three (PGB1) stages of degradation.

The main stages of degradation for PNVI and gellan occurred in the temperature ranges 406–519 °C and 241–322 °C, respectively being characterized by an important weight loss of 47.30% and 44.96%, probably due to the degradation of the polymer backbone. The degradation results of PG showed that the grafting of NVI onto gellan gum increased the thermal stability of the copolymer, the main stage of decomposition occurring in the range 429–548 °C with a weight loss of 15.85%. This behavior can be explained by the presence of an imidazole ring belonging to PNVI which is thermally stable. The same observations were reported by other authors for grafting of poly(*N*-vinylimidazole) onto carboxymethyl chitosan [32]. The introduction of betaine units led to decrease of thermal stability of PGB1, the main stage of decomposition taking place in the range 256–410 °C with a weight loss of 40.92%. After thermal treatment up to 700 °C the remaining residual masses were 3.92% (PNVI), 16.14% (GLL), 53.29% (PG) and 31.18% (PGB1), respectively. Taking into account the results previously presented, it can be said that the grafting reaction was realized and the grafting of NVI onto gellan gum led to an increase in thermal stability of the PG copolymers, whereas the introduction of betaine units induced a slight decrease in the thermal stability of PGB1 copolymers.

### 2.5. Immobilization and Drug Release

The synthesis of grafted copolymers with *N*-vinyl imidazole (PG), and subsequently functionalized with betaine structure (PGB1), was made in order to obtain precursors capable of binding amphoteric drugs through ionic interactions. Consequently, a preliminary study was performed on the immobilization; respectively the release of an amphoteric model drug (cefotaxime sodium salt) to verify our hypothesis. The ionic interaction of the drug is possible both with the copolymer PG, which contains carboxylic groups belonging to gellan, and especially with the copolymer PGB1, which contains carboxylic groups belonging to both the gellan and the betaine structure.

Cefotaxime sodium salt (CF) is a semisynthetic third generation cephalosporin with bactericidal activity, being more active against gram-negative bacteria than gram-positive bacteria [44].

Cefotaxime sodium salt was immobilized onto PG and PGB1 samples in a bach system. The maximum immobilization capacities of CF onto PG and PGB1 samples were found to be 420 mg/g and 473.2 mg/g, repectively. Obviously, the higher CF binding capacity was higher for the PGB1 copolymer which contains a higher number of carboxylic groups compared to the PG copolymer.

**Figure 14:**
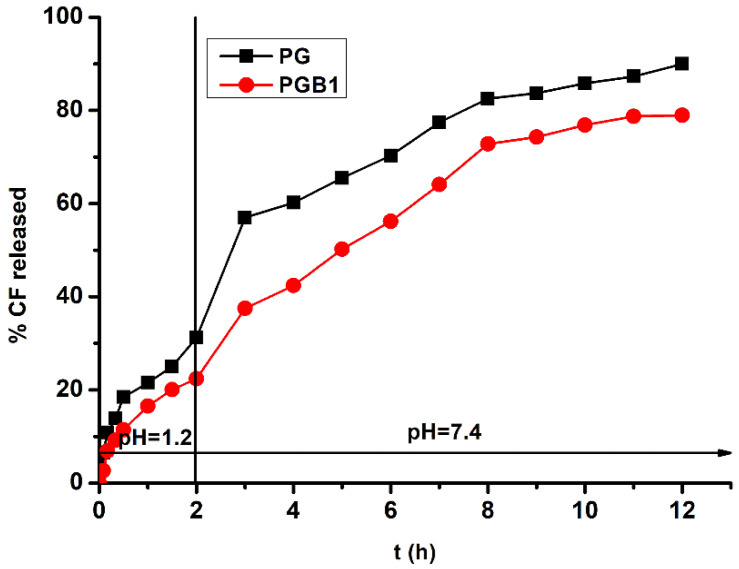
Release profile of cefotaxime sodium salt from PG and PGB1 samples.

The release profiles of CF as a function of time are presented in Figure 14.

From Figure 14, it can be observed that the amount of CF released from PG and PGB1 was higher at pH = 7.4 than pH = 1.2. In acidic pH values, the carboxylate groups of gellan became protonated, the repulsions between polymer chains being much diminished resulting in a decrease of swelling degree and finally in a decrease of the amount of drug released. At higher pH values, the carboxylate groups were ionized and the electrostatic repulsive forces between COO^−^ groups located on the copolymer backbone caused an increase of the swelling capacity leading to an increase of amount of drug released.

To analyze the type of release mechanism, the drug release data were fitted using two release kinetic models: the Higuchi and Korsmeyer-Peppas models.

The Higuchi model is based on the Fick’s law and is used to describe the release of a water soluble drug from solid matrices [45]. The mathematical equation of Higuchi model is:

(10) Qt=kH·t12

where *k_H_* is Higuchi dissolution constant.

The Korsmeyer-Peppas model describes the mechanism of drug release from polymeric systems [46]:

(11) $$\frac{M_{t}}{M_{\infty }}=k_{r}\cdot t^{n}$$

where *Mt*/*M*_∞_ = fraction of drug released at time *t*; *k_r_* = release rate constant that is characteristic to polymer-drug interactions; *n* = the diffusion exponent is characteristic to the different release mechanisms.

Depending on the values of n, the release can take place through several mechanisms such as: *n* = 0.5, Case I diffusion or Higuchi kinetic (diffusion/controlled drug release); 0.5 < *n* < 1, anomalous diffusion; *n* = 1, Case II transport (swelling-controlled drug release); *n* > 1, super Case II transport.

The values of the release parameters of CF from PG and PGB1 are presented in Table 2.

Based on the release exponent n from the Korsmeyer-Peppas equation, the release mechanism of CF from the PG sample is controlled by a diffusion process while the release mechanism of CF from the PGB1 sample is more complex, being controlled by both swelling and diffusion processes.

The results presented in this study attest the ability of the two types of copolymers to bind amphoteric drugs through electrostatic interactions. An in-depth study on the immobilization of biologically active principles with cationic or anionic characteristics on grafted copolymers carrying betaine units is the subject of further work.

## 3. Materials and Methods

### 3.1. Materials

Gellan gum (M_w_ = 1 × 10^6^ g/mol), ammonium persulfate, *N*,*N*,*N*′,*N*′-tetramethylethylenediamine, acetone, ethanol, sodium chloroacetate, hydrochloric acid, potassium chloride, monobasic and dibasic sodium phosphate and cefotaxime sodium salt were supplied from Sigma-Aldrich, Germany and were used as received. *N*-vinylimidazole was purchased from Sigma Aldrich, Germany and was distilled under vacuum. Ultrapure grade water (Ω < 10^−6^ s/cm) was prepared by purifying deionized water with Millipore Simplicity-UV apparatus.

### 3.2. Synthesis of Grafted Polymers (PG)

The PG copolymers were prepared by using the free radical polymerization technique in presence of redox initiator (APS + TEMED) in nitrogen atmosphere.

60 mL of (2–10 g/L) gellan solution in ultrapure distilled water was added into a 250 mL three-necked round-bottom flask equipped with a magnetic stirrer and a thermostater water bath. To gellan solution various amounts of NVI (c_m_ = 0.3–2 mol/L) were added followed by the addition of different amounts of initiator solutions (c_i_ = 0.02–0.1 mol/L). The molar ratio between APS and TEMED was 1:1, which has been reported in literature to be the optimal value for obtaining free radicals as well as the polymer with highest grafting yield [47]. The grafting reactions were carried out at different reaction temperatures (30–80 °C) and at various periods of time (2–6 h). Finally, the grafting mixture was precipitated under vigourous stirring in cold acetone and then the grafted copolymers were separated by filtration under vacuum using borosilicate glass filter crucibles, porosity 4. Thereafter, the grafted copolymers were purified by extraction with ethanol in a Soxhlet apparatus in order to remove the homopolymer, and were dried in vacuum oven at 50 °C for 48 h.

### 3.3. Estimation of Grafting Parameters

The grafting parameters were calculated as follows [48,49].

(12) $$GY\;\left(\%\right)=\;\frac{weight\;of\;grafted\;PNVI}{weight\;of\;gellan}\cdot 100$$

(13) $$GE\;\left(\%\right)=\;\frac{weight\;of\;grafted\;PNVI}{weight\;of\;polymer\;formed}\cdot 100$$

(14) $$C\;\left(\%\right)=\;\frac{weight\;of\;polymer\;formed}{weight\;of\;monomer\;fed}\cdot 100$$

(15) H (%)=100−GE (%)

### 3.4. Synthesis of Grafted Polymer Carrying Betaine Units

Grafted polymer carrying betaine units was prepared by the betainization reaction of PG with maximum grafting yield in the presence of sodium chloroacetate. Thus, 5 g of PG were swollen in water for 24 h at room temperature then centrifuged at 78 RCF for 10 min after which an aqueous solution of sodium chloroacetate (100 mL, c = 20%, *w*/*v*) was added. To calculate the amount of betainization agent required for the polymer-analogous reaction, the molar ratio between nitrogen and sodium chloroacetate was considered to be 1:1.5. The reaction mixture was gently stirred at T = 60 °C for 72 h. The final product was removed from the reaction medium by precipitation in acetone and then the precipitate was extracted with water in a Soxhlet apparatus. The final product was insoluble in water.

### 3.5. Infrared Spectroscopy

FTIR spectra were recorded on a Bruker Vertex 70 FTIR spectrometer (Wien, Austria) at a resolution of 2 cm^−1^ in the frequency range of 400–4000 cm^−1^, by the KBr pellet technique (pellets obtained at a pressure of 2 tons for 1 min).

### 3.6. ^1^H-NMR Analysis

^1^H-NMR measurements of PNVI, gellan and PG were performed on a high-resolution liquid NMR 400 MHz spectrometer Bruker Neo-1 (Rheinsteitten, Germany) for direct detection probe, with 5 mm Quadra nuclei probes, QNP (four nuclei, ^1^H/^13^C/^19^F/^29^Si). Sample solutions were prepared with deuterated water and 1N NaOH as solvent. TSP (trimethylsilylpropanoic acid) (δ = 0.0 ppm) was used as an internal standard. Detection temperature was set at 25 °C and the sample was scanned 64 times.

### 3.7. Scanning Electron Microscopy (SEM)

The surface morphologies of gellan, PNVI, PG and PGB1 in powder form were analyzed with an environmental scanning electron microscope type Quanta 200 at 25 kV with secondary electrons in low vacuum. The microscope was coupled with an energy dispersive X-ray system for qualitative and quantitative analysis.

### 3.8. X-Ray Diffraction Analysis (XRD)

The XRD patterns of PNVI, GLL, PG and PGB1 samples were recorded employing a D8 Advance Bruker AXS device using a CuKα radiation at a current/voltage of 36 mA/30 kV.

### 3.9. Thermogravimetric Analysis (TG/DTG)

The thermal stability of the crosslinked epoxy resins were thermogravimetrically analyzed using a STA 449 F1 Jupiter apparatus (Netzsch, Selb, Germany) coupled to a Vertex 70 spectrophotometer for FT-IR analysis and Aeölos QMS 403C mass spectrometer (Netzsch-Germany) for the mass spectroscopic analysis of the evolved gases. Samples of about 10 mg placed in Al_2_O_3_ crucibles were thermally degraded at a heating rate of 10 °C min^−1^, under air atmosphere in the temperature range between 25 °C and 700 °C.

### 3.10. Immobilization and Drug Release

CF immobilization was realized as follows: 0.2 g of PG and PGB1 copolymers were weighed into 50 mL conical flasks and then 20 mL of CF (c_CF_ = 3·10^−3^ g/mL) were added. The samples were placed in a thermostatic shaker bath (Memmert MOO/M01, Schwabach, Germany) and shaken at 180 strokes/minute until equilibrium was reached. The flasks were removed from the shaker and the copolymers were centrifuged at 78 RCF for 10 min. The amount of CFR immobilized onto PG and PGB1 copolymers was determined by UV-VIS spectrophotometry (SPEKOL 1300 Spectrophotometer, Analytik Jena, Jena, Germany) at a wavelength of 236 nm, based on a calibration curve.

The amount of drug immobilized was obtained using the following equation:

(16) $$q_{e}=\frac{\left(C_{0}-C_{e}\right)\cdot V}{W}$$

where *q_e_* is the amount of CF immobilized onto PG and PGB1 copolymers (mg/g), *C*_0_ is the initial concentration of drug (mg/mL), *C_e_* is the drug concentration at equilibrium (mg/mL), *V* is the volume of drug solution (mL) and *W* is the weight of the copolymers.

In vitro release studies of cefotaxime sodium salt were performed by immersing the PG and PGB1 samples (0.1 g) in 10 mL of simulated gastric fluid (pH = 1.2) for 2 h and phosphate buffer solution (pH = 7.4) for 10 h at 37 °C. The buffer solutions were prepared according to protocols well known in the literature [50,51]. The samples were placed in a thermostated shaker bath (Memmert M00/M01, Germany) under gentle shaking (50 strokes/minute). Withdrawal of a small volume (1 μL) of release solution was done and the CF solutions collected at different intervals of time were measured spectrophotometrically at a wavelength of 236 nm using a UN-VIS spectrophotometer (Nanodrop ND 100, Wilmington, DE, USA). The amount of CF released was calculated using a calibration curve.

## 4. Conclusions

Grafted polymers carrying betaine units were obtained by grafting *N*-vinylimidazole onto a gellan gum backbone followed by a betainization reaction of the grafted copolymer with maximum grafting yield in the presence of sodium chloroacetate. The grafted copolymers were successfully obtained by a free radical polymerization technique in the presence of redox initiator (APS + TEMED) and a nitrogen atmosphere. From the point of view of the reaction mechanism, the betainization reaction is a nucleophilic reaction and the betainization degree estimated by FTIR spectroscopy was found to be about 90%. The grafting parameter (GY%, GE%, H% and C%) could be adjusted by changing one of the reaction parameters. The optimized reaction conditions for the grafting reaction of NVI onto GLL were as follows: initiator concentration = 0.08 mol/L; monomer concentration = 0.8 mol/L; polymer concentration = 8 g/L; reaction temperature = 50 °C and the reaction time = 4 h. The mechanism of grafting reaction is similar with that of free radical polymerization.

FTIR spectroscopy, ^1^HNMR spectroscopy, X-ray diffraction, thermogravimetric analysis and scanning electron microscopy confirmed the grafting reaction of NVI onto gellan, as well as the synthesis of grafted polymers having betaine structure.

In vitro release studies of CF proved the capacity of the new grafted copolymers to immobilize amphoteric drugs and highlighted the fact that the release mechanism of CF from PG and PGB1 samples is controlled by diffusion process or by a combination between diffusion and swelling processes. These results demonstrated that the grafted copolymer with betaine structure can be a potential candidate for developing sustained/controlled drug delivery systems.

## Figures and Tables

**Table 1:** Thermogravimetric analysis data for main stage of decomposition.

Sample Codes	Decomposition Temperature			Weight Loss (%)	Residual Mass (%)
	T_i_ (°C)	T_m_ (°C)	T_f_ (°C)		
PNVI	406	433	519	47.3	3.9
GLL	241	250	322	45.0	16.1
PG	429	454	548	15.9	53.3
PGB1	256	278	410	40.9	31.2

**Table 2:** Kinetic parameters of CF release from PG and PGB1.

Sample Codes	Higuchi Model		Korsmeyer-Peppas Model		
	*k_H_* (h^−1/2^)	R^2^	*k_r_* (min^−n^)	*n*	R^2^
**PG**	0.249	0.993	0.053	0.377	0.992
**PGB1**	0.236	0.990	0.023	0.550	0.991
